# Cuguillere’s Serum in the Treatment of Tuberculosis

**Published:** 1908-10

**Authors:** 


### Cuguillere's Serum in the Treatment of Tuberculosis.

Eugene Caravia, New York, describes Cuguillere's serum as a vegetable serum which carries colloidal metals already assimilated, that have become organic, living, and possessed of their maximum activity. It is a yellow liquid with an odor of garlic, and is made from organic sulphur taken from the juices of brassica, allium, watercress, horse-radish and other herbs, which are antiscorbutic and antirachitic. Its formula is: allylum sulphide 1.0; tincture of myrrh 1.1; Hayem's glycerinated serum 100. The author gives descriptions of experiments on animals and man by the use of this serum as a curative treatment in various forms of tuberculosis, undertaken by various authors in different countries. Examinations were made of the internal organs of cattle cured by this method with the result of finding no tubercle bacilli, and cure by cicatrisation and phagocytosis. The serum acts in advanced cases where tuberculin is powerless on account of the too great reaction produced. It increases appetite, facilitates digestion and increases weight. Treatment is easier than with tuberculin. The minimum dose is one to two cubic centimeters and the maximum five to fifteen. The writer reports four cases treated by himself and cured. This serum attacks the cause of the disease, the bacilli themselves. It promotes fibrosis, it stops hemorrhage, lessens fever, stops diarrhea and night sweats, helps appetite, strengthens the heart, and lessens cough and expectoration.—*Medical Record,* September 26, 1908.

